# Seasonal variation in the prevalence of *Toxocara* eggs on children’s playgrounds in the city of Hanover, Germany

**DOI:** 10.1186/s13071-017-2193-6

**Published:** 2017-05-19

**Authors:** Annika Kleine, Andrea Springer, Christina Strube

**Affiliations:** Institute for Parasitology, Centre for Infection Medicine, University of Veterinary Medicine Hannover, Buenteweg 17, 30559 Hanover, Germany

**Keywords:** *Toxocara*, Roundworms, Zoonosis, Geohelminths, Soil-transmitted helminths, Playgrounds, Sandpits, Soil, Children

## Abstract

**Background:**

Roundworms of the genus *Toxocara* are worldwide distributed zoonotic parasites of carnivores. Based on case numbers and the potential impact on human health, the Centers for Disease Control and Prevention (CDC) categorised toxocarosis as one of the most important neglected parasitic diseases. As contact with contaminated soil, e.g. in sandpits, is considered the primary transmission route, data on playground contamination are needed to assess infection risk for children. Here, playground contamination rates and their seasonal variation in the city of Hanover, Germany, were investigated.

**Methods:**

Sand samples were collected monthly over a 12-month period on 46 playgrounds in the city of Hanover, Germany. In total, 1,362 samples were examined for *Toxocara* eggs and analysed statistically for seasonal influences on potential infection risk.

**Results:**

Contamination rates ranged from 6.5% (3/46) *Toxocara* positive sandpits in September to 41.3% (19/46) in February, while contamination with infective embryonated eggs varied between 2.2% (1/46) and 23.9% (11/46). Compared to September, the month with the lowest contamination rate, significantly more sandpits were positive for *Toxocara* eggs from January to August and in October, while the prevalence of infective *Toxocara* eggs was significantly increased only in January and February. Regarding egg numbers, significantly higher total counts were observed in October and from December to June, while infective egg counts were significantly increased only in January, February and April.

**Conclusions:**

Compared to data from 1985, contamination rates have dropped from 55.8% to an average of 23.2% in 2011. Even though the observed egg numbers indicate a moderate to low general infection risk, the potential risk to single individuals should not be underestimated, as highly contaminated spots may occur infrequently and independent of season.

**Electronic supplementary material:**

The online version of this article (doi:10.1186/s13071-017-2193-6) contains supplementary material, which is available to authorized users.

## Background

*Toxocara canis* and *T. cati* are worldwide distributed zoonotic roundworms of domestic and wild canids and felids, respectively, which may cause several forms of disease in paratenic hosts, including humans. Based on the number of people infected, the potential severity of the illness and the possibility of prevention and treatment, the American Centers for Disease Control and Prevention (CDC) categorises human toxocarosis as one of the five neglected parasitic diseases with priority for public health action [1]. As a consequence of oral infection, several clinical manifestations caused by migrating larvae may arise in humans, which are classified as visceral and ocular larva migrans syndrome (VLM and OLM), covert toxocarosis and neurotoxocarosis [2]. VLM is a multi-systemic disease due to invading *Toxocara* larvae, causing fever and abdominal as well as respiratory symptoms. OLM may result in retinal granuloma with visual impairment or even loss of eyesight. Covert toxocarosis describes a non-specific clinical syndrome with varying symptoms, such as fever, headache, abdominal pain and behavioural disorders. Finally, neurotoxocarosis may cause a number of neurological dysfunctions. Recently, a link between *Toxocara* exposure and decreased cognitive performance in children has been proposed [3], which is supported by mouse models showing decreased learning ability in infected mice [4].

When excreted by carnivores as definitive hosts, *Toxocara* eggs are not immediately infective, but embryonate in the environment by developing the infective third-stage larva within the eggshell within three to six weeks under appropriate conditions [5]. Due to their sticky and thick shell, eggs adhere to environmental surfaces and may remain viable for several months under optimal conditions. Humans become infected by ingesting embryonated eggs after contact with contaminated soil or by consumption of contaminated raw vegetables and herbs. Furthermore, the infection may be acquired by ingesting infective third-stage larvae in raw or undercooked tissues of paratenic hosts (e.g. chicken). Although the relative epidemiological importance of each of these transmission pathways remains unclear, environmental contamination is regarded as the most important source of infection [6].

Human seroprevalence to *Toxocara* in continental Europe varies between 2.4% in Denmark and 44% in Austria with higher values in rural areas [7, 8]. In tropical countries, seroprevalence can be as high as 90% [9]. However, current data on seroprevalence are lacking for many countries. Due to lower hygienic awareness, frequent hand-to-mouth contact and oral exploration, including geophagy, children are especially at risk of acquiring infections. Children between five and 14 years of age were more frequently found to be *Toxocara* seropositive than other age classes [10], and a positive correlation between the frequency of playing at public squares and *Toxocara* seropositivity has been demonstrated [11]. Thus, the occurrence of *Toxocara* eggs in sandpits needs to be assessed to estimate the infection risk for children when playing on public playgrounds, and inform public health authorities with regard to the implementation of control measures. The last study investigating geohelminth prevalence on public places in Hanover was conducted in 1985 [12] and reported a contamination rate with *Toxocara* spp. of 55.8%. However, the prevalence of *Toxocara* spp. in Germany has declined from 6.9% in dogs and 9.5% in cats in the years 1984–1991 [13] to 2.8–6.1% in dogs and 3.9–4.7% during the years 2003–2012 ([14]; unpublished own diagnostic data based on 2,731 dog and 903 cat samples). Therefore, a re-evaluation of playground contamination rates with *Toxocara* spp. in the city of Hanover, Germany, seemed necessary and is provided in the presented study.

## Methods

### Study area

Hanover is the capital city of the federal state of Lower Saxony, Germany. It houses about 540,000 inhabitants with 14.8% below the age of 18 (state of data: November 9^th^, 2016, [15]). The total area of Hanover covers 20,414 ha, 13.2% of which account for public parks (state of data: January 1^st^, 2016, [15]), including 406 playgrounds. Sand of public sandpits is replaced regularly every three years with sand from gravel pits, whereas sand from other playground features, such as climbing frames is replaced every five years.

### Sample collection

From January to December 2011, sand samples were collected monthly from sandpits of 46 playgrounds. Playgrounds were chosen to represent all districts of Hanover and were distributed approximately evenly over the entire area of the city. Additionally, sand under thirteen climbing frames was sampled monthly from March to December.

Samples were taken in a meandering pattern from the surface layer of sand (maximum depth 2–3 cm) where *Toxocara* eggs are mostly found [16]. A total of five samples per sandpit (about 1 kg in total) were taken each month, which was later combined into one monthly sample per sandpit (see section below). In addition, sandpits and playgrounds were surveyed for carnivore faeces which were collected and examined by the McMaster method [17].

### Preparation of sand samples

To ensure comparable sample weights, samples were dried at 37 °C. The drying time depended on the original degree of moisture, which was determined by drying a sample subset at 105 °C according to the Darr method [18]. After drying, the five samples taken from each sandpit per month were mixed in a side sealed bag (Pelle Vakuumverpackung, Spelle, Germany) to obtain one homogenous sample per month.

Of each sample, two fractions of 250 g each were treated by the egg recovery method as described by Kleine et al. [19]. Briefly, sample fractions were mixed with 250 ml of saturated sodium chloride (NaCl) solution containing 0.2% Tween 80® (Carl Roth GmbH, Karlsruhe, Germany). After incubation for 10 min in a moving water bath and 20 min on a distributing jigger, samples were transferred to a 1 l beaker. Saturated NaCl solution was added to a total volume of 750 ml, and potentially incorporated eggs were allowed to float to the surface for one h. The supernatant was drained through a 25 μm sieve; the content of the sieve was transferred by tap water to a 50 ml tube and centrifuged at 3000× *g* for 10 min. The sediment was microscopically examined for helminth eggs.

### Statistical analysis

Statistical analyses were conducted in R v. 3.3.1 [20]. Generalised linear mixed models (GLMMs) with binomial error structure and logit link function were used to test the effect of sampling month on the likelihood of sandpit contamination with *Toxocara* eggs. Models were implemented using the R package lme4 [21]. To account for repeated sampling, playground identity was included as a random factor. Monthly variation regarding the egg numbers per sandpit sample was assessed in GLMMs with negative binomial error structure and log link function because of zero-inflated, overdispersed data, using lme4’s “glmer.nb”-function. Negative binomial GLMMs also included playground ID as a random factor. Full models were compared to null models containing only the random factor in a likelihood ratio test (R-function “ANOVA", method = “chisq”). Additional models were run including results from sampling of climbing frames.

## Results

### Faecal examination

In total, 31 carnivore faecal samples were found over the year on 15 of the 46 playgrounds, 26 of which were attributed to dogs and five to cats. Only one dog sample contained eggs of *Toxocara* spp. and *Trichuris vulpis*.

### Playground contamination with zoonotic helminth eggs

**Fig. 1:**
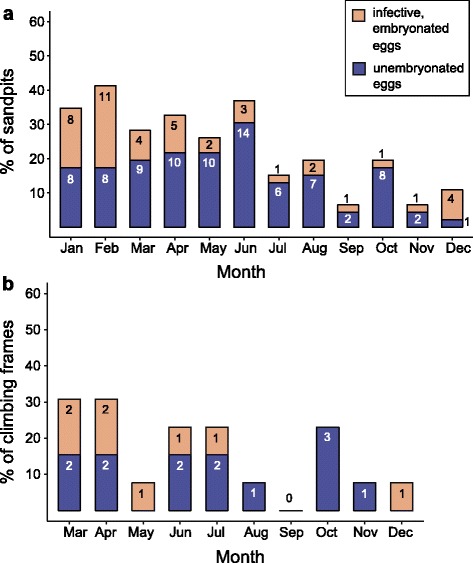
Monthly contamination rates of (**a**) sandpits (*n* = 46) and (**b**) climbing frames (*n* = 13) with *Toxocara* eggs on the sampled 46 playgrounds in the city of Hanover, Germany. The bars indicate the number of sandpits; the Y-axis indicates the percentage ratio

Overall, the percentage of sandpit samples positive for *Toxocara* eggs was 23.2%, with monthly contamination rates varying between 6.5% (3/46) in September and 41.3% (19/46) in February. Embryonated infective *Toxocara* eggs were found in 7.8% of sandpit samples, ranging from 2.2% (1/46) in July, September, October and November to 23.9% (11/46) in February. Detailed data on monthly contamination rates of sandpits and climbing frames are shown in Fig. 1.

**Fig. 2:**
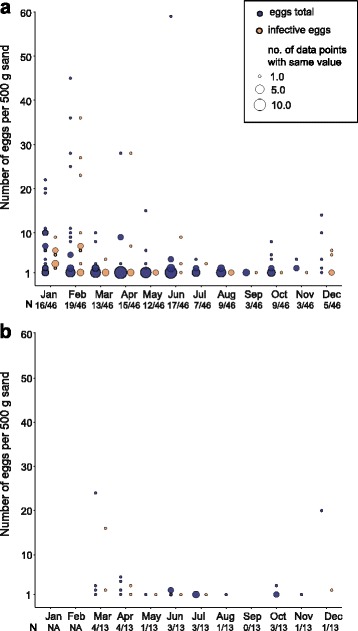
Monthly variation of *Toxocara* eggs in 500 g sand from (**a**) sandpits (*n* = 46) and (**b**) climbing frames (*n* = 13) of contaminated playgrounds. The number of positive samples in each month is indicated below the X-axis. Circle size is proportional to the number of data points with the same value. *Abbreviation*: NA, not applicable (not sampled)

The average number of *Toxocara* eggs per playground varied from 0 to 5.9 eggs in 500 g of sand over the 1-year study period. In positive samples, 1 to 59 eggs (mean: 4.9, SD: 8.6) were observed. Regarding infective eggs, average numbers on positive playgrounds ranged between 0 and 3.58 eggs per 500 g of sand, with a minimum of 1 egg and a maximum of 36 eggs (mean 1.4, SD: 4.9). Data on total and infective egg numbers on playgrounds are displayed in Fig. 2. Neither hookworm or whipworm eggs nor *Ascaris* spp. eggs were detected in any sand sample.

### Seasonal *Toxocara* egg contamination

Overall, the likelihood of sandpit contamination with *Toxocara* eggs was significantly higher from January to June when compared to September, the month with the lowest contamination rate (Table 1; GLMM, likelihood ratio test: *χ* ^2^ = 44.4, *df* = 11, *P* < 0.001). Sandpit contamination with infective eggs was observed significantly more often in January and February as compared to September (Table 1; GLMM, likelihood ratio test: *χ* ^2^ = 29.9, *df* = 11, *P* = 0.002). The likelihood of contamination did not differ significantly between sandpits and climbing frames (Additional file 1: Table S1).

**Table 1:** Results of GLMMs with binomial error structure testing monthly differences in contamination rates with total and embryonated infective *Toxocara* eggs in playground sandpits

	Term	Estimate	Std. Error	*z*-value	*P*-value
Total contamination with *Toxocara* eggs	Intercept	-2.70	0.60	-4.49	< 0.001***
	January	2.09	0.68	3.09	0.002**
	February	2.34	0.67	3.49	< 0.001***
	March	1.75	0.68	2.56	0.01*
	April	1.96	0.68	2.89	0.004**
	May	1.64	0.69	2.38	0.017*
	June	2.16	0.67	3.20	0.001**
	July	0.95	0.73	1.31	0.190
	August	1.26	0.71	1.79	0.074
	October	1.26	0.71	1.79	0.074
	November	0.00	0.85	0.00	0.999
	December	0.56	0.76	0.74	0.462
Contamination with embryonated infective *Toxocara* eggs	Intercept	-3.81	1.01	-3.77	< 0.001***
	January	2.28	1.08	2.10	0.036*
	February	2.68	1.07	2.51	0.012*
	March	1.46	1.14	1.28	0.201
	April	1.70	1.12	1.53	0.127
	May	0.72	1.24	0.58	0.565
	June	1.14	1.17	0.97	0.330
	July	0.00	1.43	0.00	1.000
	August	0.72	1.24	0.58	0.565
	October	0.00	1.43	0.00	1.000
	November	0.00	1.43	0.00	1.000
	December	1.46	1.14	1.28	0.201

*P ≤ 0.05, **P ≤ 0.01, ***P ≤ 0.001

September, the month with the lowest contamination rate, was chosen as reference. Asterisks indicate statistically significant differences

Regarding egg numbers, sandpit samples contained significantly more *Toxocara* eggs in October and from December to June as compared to September (Table 2, GLMM, likelihood ratio test: *χ* ^2^ = 65.89, *df* = 11, *P* < 0.001). The number of infective eggs per 500 g of sand was significantly higher in January, February and April (Table 2, GLMM, likelihood ratio test: *χ* ^2^ = 858.15, *df* = 11, *P* < 0.001). Again, there was no statistically significant difference between egg numbers in sandpit compared to climbing frame samples (Additional file 2: Table S2).

**Table 2:** Results of GLMMs with negative binomial error structure testing the effect of month on the number of total and embryonated infective *Toxocara* eggs per sandpit sample

	Term	Estimate	Std. Error	*z*-value	*P*-value
Total number of *Toxocara* eggs	Intercept	-2.83	0.71	-3.99	< 0.001***
	January	3.83	0.81	4.73	< 0.001***
	February	4.23	0.80	5.26	< 0.001***
	March	2.47	0.82	3.01	0.003**
	April	2.89	0.82	3.53	< 0.001***
	May	2.44	0.83	2.96	0.003**
	June	3.04	0.82	3.70	< 0.001***
	July	1.30	0.86	1.52	0.128
	August	1.49	0.85	1.74	0.082
	October	2.04	0.83	2.46	0.014*
	November	0.92	0.88	1.05	0.294
	December	2.17	0.84	2.60	0.009**
Number of embryonated infective *Toxocara* eggs	Intercept	-4.06	1.18	-3.43	< 0.001***
	January	3.74	1.32	2.83	0.005**
	February	4.89	1.32	3.71	< 0.001***
	March	1.90	1.37	1.39	0.164
	April	3.27	1.36	2.41	0.016*
	May	0.57	1.49	0.38	0.704
	June	2.04	1.38	1.47	0.141
	July	0.60	1.49	0.40	0.687
	August	0.71	1.48	0.48	0.631
	October	-0.30	1.68	-0.18	0.858
	November	-0.07	1.64	-0.04	0.965
	December	2.36	1.35	1.75	0.081

*P ≤ 0.05, **P ≤ 0.01, ***P ≤ 0.001

September, the month with the lowest contamination rate, was chosen as reference. Asterisks indicate statistically significant differences

### Discussion

Zoonotic roundworms of the genus *Toxocara* pose a threat to public health due to the clinical manifestations they may cause in humans, such as visceral and ocular larva migrans syndrome or neurotoxocarosis. The high seroprevalences reported globally highlight the need for contemporary studies assessing transmission risks originating from different reservoirs. It has been suggested that environmental transmission is the main source of infection for humans [6]. In children, a positive correlation between frequency of playing at contaminated public places and *Toxocara* seropositivity has been demonstrated [10, 11]. Here, public playgrounds were surveyed for *Toxocara* eggs in the city of Hanover, Germany.

Monthly *Toxocara* contamination rates of sandpits ranged from 6.5% in September to 41.3% in February. Such range is comparable to current contamination rates found in Belgium and Poland [22, 23]. By contrast, Horn et al. [12], who used a similar egg detection method, found 55.8% of Hanoverian playgrounds to be contaminated with *Toxocara* eggs in August 1985. The apparent decrease in contamination rates may be attributed to the reduction in *Toxocara* prevalence in dogs and cats. Between 1984 and 1991, 6.9% of dogs and 9.5% of cats in Germany excreted *Toxocara* eggs with their faeces [13]. Between 2003 and 2012, these numbers dropped to 2.8–6.1% in dogs and 3.9–4.7% in cats [14; unpublished own diagnostic data based on 2,731 dog and 903 cat samples], probably due to improved awareness for parasites and anthelmintic management by veterinarians and pet owners. Similarly, only one out of 31 faecal samples found on playgrounds in the present study was positive for parasite eggs, including *T. canis*. Furthermore, only low average numbers of *Toxocara* eggs were detected in positive sand samples (4.9 total eggs/500 g of sand, 1.4 infective eggs/500 g of sand). In comparison, a number of 2.1 viable eggs per five grams of soil is considered as a high risk for human infection [24]. Thus, the infection risk for children playing on Hanoverian playgrounds is estimated to be moderate to low. However, because low infection doses may not stimulate the immune system adequately, they are considered to be the main cause of ocular toxocarosis [25]. Therefore, the risk for a single individual of acquiring *Toxocara* infections from sandpits should not be underestimated, as individual sand samples contained up to 36 infective eggs per 500 g, probably due to recent contamination with faeces.

Over the course of the year, significant seasonal variation in *Toxocara* contamination rates as well as in egg numbers was found, indicating that infection risk may vary depending on the season. Percentage contamination rates were elevated from January to June, with January and February showing the highest contamination rate with infective eggs. Similarly, the number of *Toxocara* eggs per sandpit sample was elevated from December to June, and highest numbers of infective *Toxocara* eggs were again found in January and February. Higher percentages of *Toxocara* contamination of playgrounds and public parks in winter and spring than in summer and autumn were also reported in other studies from Europe [23, 26], demonstrating a general pattern underlying this observation. Survival and development of *Toxocara* eggs depend on temperature, humidity and sunshine exposure [27, 28]. Development starts around 4 °C and accelerates with ascending temperatures, but as of 37 °C, eggs are damaged. Development also increases with humidity, whereas during arid conditions, *Toxocara* eggs arrest development or die. The cool and wet winter weather in central Europe may facilitate egg survival over prolonged periods, resulting in accumulation of contamination during the first half of the year, whereas sunshine exposure and desiccation of sand during summer and autumn are a relevant cause for declining contamination during these seasons. Furthermore, dog owners may have stronger inhibitions to let their dogs run on playgrounds during summer months, when these are frequently visited by children. Additionally, seasonal patterns of *Toxocara* prevalence and egg shedding in the definitive host may cause seasonal variation in contamination rates. In dogs, patent *T. canis* infections peak during wintertime, possibly related to the reproductive biology of the host [29]. In the present study, eggs of *T. canis* and *T. cati* could not be microscopically differentiated, and a PCR-based approach was unsuccessful, probably due to UV-degradation of DNA (unpublished results)*.* In a numerical model which estimated the contribution of different host species to environmental *Toxocara* contamination in Bristol, UK, dogs were predicted to shed the majority of *Toxocara* eggs into the environment, at least when removal of faeces by dog owners was neglected [30]. However, this study did not take differences between host species with regard to defecation habits into account. Therefore, it may be suggested that a significant proportion were *T. cati* eggs as shown on Japanese sandpits [31] and in urban areas of Poland [26], as cats prefer sandy substrates for defecation rather than dogs. Building upon the model by Morgan et al. [30], Nijsse et al. [32] suggested cats to be the main source of *Toxocara* egg contamination in urban areas in the Netherlands. Furthermore, contamination by wildlife needs to be considered. Urban fox populations, which display high *T. canis* prevalences, have been on the rise all over Europe and may represent a non-negligible reservoir [33]. For example, 59% of foxes were found to be infected with *Toxocara canis* in Denmark [34]. In the numerical model mentioned above, foxes were predicted to constitute the main source of *Toxocara* eggs in Bristol, UK, at high levels of faeces removal by dog owners [30]. Similar to dogs, foxes display a peak in *Toxocara* prevalence during the reproductive period in spring [33]. Remarkably, no hookworm eggs or any other zoonotic geohelminths apart from *Toxocara* were found on the playgrounds. However, the used method for egg recovery from sand [19] is only evaluated for *Toxocara* eggs, and hookworm larvae may have hatched from eggs during desiccation of sand samples.

To reduce *Toxocara* contamination of sandpits, fencing of playgrounds has proven effective in some studies [35], whereas other measures, like covering sandpits overnight, may not be feasible on public playgrounds. Considering that *Toxocara* eggs may accumulate over time, the interval in which sand is replaced might also have an influence on contamination. In the city of Hanover, sand in sandpits is regularly replaced every three years, whereas the replacement interval for sand underneath climbing frames is five years. Therefore, a higher contamination rate of climbing frames than sandpits was expected. However, contamination rates, as well as egg numbers of climbing frame samples, did not differ from sandpit samples, indicating that in this case decontamination was mainly due to dehydration and UV-exposure. Shorter sand replacement intervals, e.g. at the end of winter when playground contamination rates are high, would further decrease *Toxocara* contamination. However, this would be logistically challenging and costly to implement. Alternatively, complete removal of sandpits from public parks might be considered.

## Conclusions

Even though *Toxocara* contamination of playgrounds in the city of Hanover has decreased since 1985, infective *Toxocara* eggs are still present on up to almost every fourth playground. Contamination rates were elevated from January to June, and the highest proportion of infective embryonated eggs was observed in January and February. On average, the number of eggs per 500 g of sand was low, indicating a moderate to low infection risk for children. However, highly contaminated spots may occur infrequently and independent of season, constituting a potential source of infection which should not be underestimated. If sandpits are maintained on playgrounds, public health authorities should implement shorter sand replacement intervals and promote the education of the public regarding the risk of zoonotic *Toxocara* infections.

## Additional files

Additional file 1: Table S1. Results of binomial GLMMs testing the influence of sampling month as well as sample type (sandpit *vs* climbing frame) on the contamination rate with *Toxocara* eggs in total as well as embryonated eggs. September, the month with the lowest contamination rate, was chosen as reference. Asterisks indicate statistically significant differences (*P ≤ 0.05, **P ≤ 0.01, ***P ≤ 0.001). Likelihood ratio tests indicated that both models were significantly different from null models (*χ* ^2^ = 55.87, *df* =12, *P* < 0.001 and *χ* ^2^ = 35.93, *df* =12, *P* < 0.001, respectively). (DOCX 19 kb)

Additional file 2: Table S2. Results of negative binomial GLMMs testing the influence of sampling month as well as sample type (sandpit *vs* climbing frame) on the total number of *Toxocara* eggs as well as the number of embryonated eggs. September, the month with the lowest contamination rate, was chosen as reference. Asterisks indicate statistically significant differences (*P ≤ 0.05, **P ≤ 0.01, ***P ≤ 0.001). Likelihood ratio tests indicated that both models were significantly different from null models (*χ* ^2^ = 80.18, *df* =12, *P* < 0.001 and *χ* ^2^ = 53.91, *df* =12, *P* < 0.001, respectively). (DOCX 18 kb)

## Abbreviations

GLMMs: Generalised linear mixed models
OLM: Ocular larva migrans syndrome
VLM: Visceral larva migrans syndrome
