# Supplementary material for: Seasonal variation in the prevalence of Toxocara eggs on children’s playgrounds in the city of Hanover, Germany
Source: Parasit Vectors. 2017 May 19;10:248. doi: 10.1186/s13071-017-2193-6 (PMC5437484; doi:10.1186/s13071-017-2193-6)
Supplement: Supplementary file 1 — Results of binomial GLMMs testing the influence of sampling month as well as sample type (sandpit vs climbing frame) on the contamination rate with Toxocara eggs in total as well as embryonated eggs. September, the month with the lowest contamination rate, was chosen as reference. Asterisks indicate statistically significant differences (*P ≤ 0.05, **P ≤ 0.01, ***P ≤ 0.001). Likelihood ratio tests indicated that both models were significantly different from null models (χ 2 = 55.87, df =12, P < 0.001 and χ 2 = 35.93, df =12, P < 0.001, respectively). (DOCX 19 kb) [file 13071_2017_2193_MOESM1_ESM.docx]

**Additional file 1**

**Table S1:** Results of binomial GLMMs testing the influence of sampling month as well as sample type (sandpit vs. climbing frame) on the contamination rate with *Toxocara* eggs in total as well as embryonated eggs. September, the month with the lowest contamination rate, was chosen as reference. Asterisks indicate statistically significant differences (* *P* ≤ 0.05, ***P* ≤ 0.01, ****P* ≤ 0.001). Likelihood ratio tests indicated that both models were significantly different from null models (*χ*² = 55.87, *df* =12, *P* < 0.001 and *χ*² = 35.93, *df* =12, *P* < 0.001, respectively).

|  | **Term** | **Estimate** | **Std. Error** | **z value** | ***P*-value** | |
| --- | --- | --- | --- | --- | --- | --- |
| **Total contamination with *Toxocara* eggs** | Intercept | -3.31 | 0.65 | -5.09 | <0.001 | *** |
|  | January | 2.32 | 0.68 | 3.44 | 0.001 | *** |
|  | February | 2.58 | 0.67 | 3.85 | <0.001 | *** |
|  | March | 2.07 | 0.66 | 3.13 | 0.002 | ** |
|  | April | 2.24 | 0.66 | 3.40 | 0.001 | *** |
|  | May | 1.70 | 0.67 | 2.52 | 0.012 | * |
|  | June | 2.32 | 0.66 | 3.52 | < 0.001 | *** |
|  | July | 1.36 | 0.69 | 1.98 | 0.048 | * |
|  | August | 1.36 | 0.69 | 1.97 | 0.048 | * |
|  | October | 1.59 | 0.68 | 2.35 | 0.019 | * |
|  | November | 0.31 | 0.79 | 0.39 | 0.696 |  |
|  | December | 0.76 | 0.73 | 1.03 | 0.301 |  |
|  | Type (ref. sandpit) | 0.36 | 0.29 | 1.23 | 0.218 |  |
| **Contamination with embryonated *Toxocara* eggs** | Intercept | -3.92 | 1.06 | -3.71 | <0.001 | *** |
|  | January | 2.57 | 1.09 | 2.37 | 0.018 | * |
|  | February | 2.97 | 1.07 | 2.78 | 0.006 | ** |
|  | March | 1.88 | 1.10 | 1.72 | 0.086 |  |
|  | April | 2.06 | 1.09 | 1.89 | 0.058 |  |
|  | May | 1.13 | 1.17 | 0.97 | 0.332 |  |
|  | June | 1.44 | 1.13 | 1.27 | 0.204 |  |
|  | July | 0.71 | 1.24 | 0.57 | 0.566 |  |
|  | August | 0.71 | 1.24 | 0.57 | 0.566 |  |
|  | October | 0.00 | 1.43 | 0.00 | 1.000 |  |
|  | November | 0.00 | 1.43 | 0.00 | 1.000 |  |
|  | December | 1.68 | 1.11 | 1.51 | 0.130 |  |
|  | Type (ref. sandpit) | -0.18 | 0.43 | -0.42 | 0.674 |  |
